# Social support score in patients with malignant diseases—with sociodemographic and medical characteristics

**DOI:** 10.3389/fpsyg.2023.1160020

**Published:** 2023-06-01

**Authors:** Snezana Corovic, Veroljub Vucic, Olgica Mihaljevic, Jelena Djordjevic, Sofija Colovic, Snezana Radovanovic, Svetlana Radevic, Ivana Simic Vukomanovic, Katarina Janicijevic, Marija Sekulic, Svetlana Djukic, Vladimir Vukomanovic, Ognjen Djordjevic, Gordana Djordjevic, Olivera Milovanovic

**Affiliations:** ^1^Faculty of Medical Sciences, University of Kragujevac, Kragujevac, Serbia; ^2^Health Center Trstenik, Trstenik, Serbia; ^3^Department of Pathophysiology, Faculty of Medical Sciences, University of Kragujevac, Kragujavac, Serbia; ^4^Department of Communication Skills, Ethics and Psychology, Faculty of Medical Sciences, University of Kragujevac, Kragujevac, Serbia; ^5^Department of Social Medicine, Faculty of Medical Sciences, University of Kragujevac, Kragujevac, Serbia; ^6^Department of Hygiene and Ecology, Faculty of Medical Sciences, University of Kragujevac, Kragujevac, Serbia; ^7^Department of Internal Medicine, Faculty of Medical Sciences, University of Kragujevac, Kragujevac, Serbia; ^8^Department of Nuclear Medicine, Faculty of Medical Sciences, University of Kragujevac, Kragujevac, Serbia; ^9^Depatment of Epidemiology, Faculty of Medical Sciences, University of Kragujevac, Kragujevac, Serbia; ^10^Department of Pharmacy, Faculty of Medical Sciences, University of Kragujevac, Kragujevac, Serbia

**Keywords:** tumors, social-, support, patients, score

## Abstract

**Introduction:**

Social support as a complex construct has a positive influence not only on a patient’s condition but also on the process of the patient’s emotional adjustment to cancer. The goal of this study is to investigate aspects of the level of social support in oncology patients and its interconnection with sociodemographic and medical variables.

**Method:**

The study was conducted as a prospective observational study in 2020, including 250 patients aged 19 and over, both sexes, with a diagnosis of oncological disease. The research was conducted in the Department of General Medicine of the Health Center Trstenik, Central Serbia, after approval by the Ethics Committee of the Health Center Trstenik, Central Serbia. A social support assessment questionnaire (Oslo-3 Social Support Scale) was used as a research instrument.

**Results:**

Data collected from the entire study population showed that bad social support was present in almost 90% of cases. Univariate and multivariate regression analysis showed a statistically significant influence of the following variables on the bad social support: education level, activity limitation, difficulties in performing daily activities, the impact of pain on the performance of activities, the need for additional help with activity, the need for help at home, unfulfilled needs for health care, means of information, anxiety score and depression score.

**Conclusion:**

Interventions to increase social support may be important for enhancing mental health and quality of life in cancer patients.

## Introduction

Social support is an interactive construct, an interpersonal transaction that occurs between those who need help and those who give the support. Most authors classify social support into three types: emotional support (when a person feels loved and has a person nearby whom he or she can trust), instrumental support (when a person has someone who can provide help in emergencies), and informational support (when he or she receives information or consultation) (Ruiz-Rodríguez et al., 2022).

According to the National Cancer Institute (NCI) definition, social support is a network of family members, friends, neighbors, and community members who provide cancer patients with psychological, physical, and financial support when they need it. Studies have shown that social support has a positive effect on cancer patient’s physical health, emotional state, well-being, and survival (Website National Cancer Institute, 2009). A cancer diagnosis has a significant impact and consequences for the patient and family. Most cancer patients successfully adjust to cancer diagnosis and treatment, but some initially struggle with a bad mood, feelings of vulnerability, sadness, and anxiety, which are usually followed by inability, weakness, depression, trauma, panic, and worries about existential survival. These feelings and concerns interfere with their normal functioning in daily activities and their quality of life (Daré et al., 2019). Knowing that they can count on the help and support of family and friends plays an important role in coping with the stress caused by disease diagnosis and treatment (Ruiz-Rodríguez et al., 2022). Social support as a complex system of different types of help has a huge constructive impact on patients’ well-being and emotionally stable acceptance of cancer diagnosis (Comijs et al., 2015; Chiu et al., 2017). It is important for cancer patients and is one of the most important psychosocial factors in cancer patients (Geue et al., 2019). Studies have shown that cancer patients who have higher levels of these kinds of support and social bonding have a better quality of life and lower mortality rates. At the same time, those who do not have all these types of support have poorer oncologic outcomes, a higher prevalence of cancer progression, and a lower overall survival rate (Mitchell et al., 2011; Lu et al., 2016; Zhu et al., 2018).

However, little is known in our country about the unmet need for social support among people with cancer and the factors associated with it. Therefore, the goal of our research was to investigate the level of social support in cancer patients and its correlation with sociodemographic and medical variables.

## Materials and methods

### Study design

The research was conducted in the form of a prospective observational study.

### Population under study

The studied population comprised 250 users of health care at the Health Center Trstenik, Central Serbia, aged 19 and over, both sexes, with a diagnosis of oncological disease. The study was conducted between July 2020 and December 2020. The study was conducted by general doctors at the Health Center Trstenik, Central Serbia.

### Sampling

Using the statistical program G*Power for the chi-square (χ2) test, with the accepted values of the probable error of the first type α = 0.05 and the power of the study of 0.95, the total sample size was estimated at 250 subjects. The sample size was calculated according to data from studies of similar design (Geue et al., 2019). The sampling method was randomized population sample. The studied population comprised users of health care at the Health Center Trstenik, Central Serbia.

The protocols of this research were approved by the Ethics Committee of the Health Center Trstenik, Central Serbia (N^o^ 759/1 of 26.06.2020.). The study adhered to the ethical standards in line with the international (Helsinki Declaration) and national legislation. In addition, the privacy of the respondents and confidentiality of data were ensured by undertaking all necessary steps in line with the Law on the Protection of Personal Data (“Official Gazette of the RS” no. 97/08, 104/09), Law on Official Statistics (“Official Gazette of the RS” no. 104/09).

Participation in the research was voluntary. Before the start of the study, patients were introduced to the purpose and procedure of the study and gave informed consent to participate in this study. Inclusion criteria for participation in the study were patients with diagnosed oncological diseases who signed informed consent for participation in the study. Data on the presence of oncological diseases as well as the presence of other chronic non-communicable diseases (comorbidity), were collected by inspecting the medical documentation (health records), while exclusion criteria were patients under 19 years of age, the presence of psychiatric illness, the presence of acute infectious (infectious) disease, the presence of chronic infectious diseases, pregnant women and patients who did not give a written consent to participate in the study.

### Assessment instruments

In addition to the general questionnaire on demographic and socio-economic characteristics (European Health Survey Questionnaire - Second Wave) (Eurostat, 2013), a questionnaire for assessing social support (Oslo-3 Social Support Scale) was used as a research instrument. The social support score (Oslo-3 Social Support Scale) was formed on the basis of three questions from the questionnaire: the first question “How many people are so close to you that you can count on them when you have serious personal problems?” [number of points from 1 (“None”) to 4 (“6 or more”)]; the second question “How much are people really interested in you, in what you do, what happens in your life?” [number of points from 1 (“They are not interested at all”) to 5 (“They are very interested”)] and the third question. “How easy is it to get practical help from neighbors if you need it?” [number of points from 1 (“Very difficult”) to 5 (“Very easy”)]. After collecting points, social support points were formed: strong social support (12–14 points), moderate (9–11 points) and bad (3–8 points) (Sarason et al., 1983). A research instruments to assess depressive and anxiety symptoms is used PHQ-9 (The Patient Health Questionnaire) questionnaire (Beck and Steer, 1990) and the Beck Anxiety Scale (BAI) (Kroenke et al., 2001).

The independent variables in the research were: sociodemographic characteristics (age, gender, family structure, marital status, education, material status, type of settlement, occupation, use of primary and hospital health care, unmet health care needs); determinants of health (alcohol and smoking use, hygiene habits, physical activity, eating habits, self-assessment of health, stress, ability to perform daily activities, presence of another chronic non-communicable disease), while the dependent variable in the study was social support in oncology patients.

### Statistical analysis

Descriptive statistics methods were used to present the data: tabulation and graphical representation. Analysis of Variance (ANOVA) used to analyze the difference between the means of more than two groups. Independent sample t-test is used to analyze the mean comparison of two independent groups. The relationships between the dependent variable (social support) and the set of independent variables were examined by univariate and multivariate logistic regression. The risk was assessed using the size of the OR (odds ratio), with a 95% confidence interval. All results where the probability is less than 5% (*p* < 0.05) were considered statistically significant. All statistical calculations were done using the commercial, standard software package SPSS, version 20.0 (Chicago, IL, United States).

## Results

The study included 250 patients diagnosed with cancer. All patients were aged 19 and over. Two thirds of the patients were women (69.6%), more often married 68.4%, with secondary education 39.6%. Based on the answers fto the questions from the questionnaire for the assessment of social support (Oslo-3 Social Support Scale) a social support score was obtained, the average value of which for the entire study population was (7.06 ± 1.25). The obtained values at the level of the entire study population point to poor social support in nearly 90% of cases. Sociodemographic and health characteristics of the respondents as well as differences in social support scores are shown in Tables 1, 2. In relation to gender, the mean value of the social support score for women was (6.94 ± 1.29), and for men (7.32 ± 1.12). Unmarried respondents, with less education and unemployed have a higher average social support score (Table 1). Major difficulties in performing daily activities (7.32 ± 0.99), severe limitation (7.37 ± 1.14), more than five chronic diseases/conditions in addition to cancer in 12 months (7.11 ± 1.36) were associated with higher average scores of social support. In relation to the stage of the disease, the existence of a statistically significant difference in the mean values of the social support score was not confirmed (*p* = 0.912), patients in the first stage 7.03 ± 1.25, patients in the second stage 7.11 ± 1.29, patients in in the third stage of the disease 7.0 ± 0.0. No statistically significant influence of the type of therapy on the average values of the social support score was shown (*p* = 0.963). Between operated and non-operated patients and the average score of social support no statistically significant difference (6.99 ± 1.32 vs. 7.14 ± 1.6) (*p* = 0.348), (Table 2).

**Table 1:** Sociodemographic characteristics of cancer patients and social support score.

Variables		*N*° (%)	Social support score	*p*
Gender	Men	76 (30.4)	7.32 ± 1.12	0.328
	Women	174 (69.6)	6.94 ± 1.29	
Marital status	Unmarried	5 (2.0)	7.40 ± 1.81	0.371
	Married	171 (68.4)	6.96 ± 1.32	
	A widower/a widow	50 (19.9)	7.26 ± 1.01	
	Divorced	24 (9.7)	7.25 ± 1.07	
Education	Elementary school	92 (36.5)	7.22 ± 1.24	0.001
	High school	99 (39.6)	7.23 ± 1.23	
	College	59 (23.6)	6.52 ± 1.17	
Employment status	Work for pay	11 (4.4)	6.00 ± 1,54	0.906
	Unemployed	58 (23.2)	7.27 ± 1.28	
	Pension	72 (28.8)	7.05 ± 1.09	
	Unable to work	26 (10.4)	6.96 ± 1.04	
	Housewife	83 (33.2)	7.08 ± 1.33	
Cigarette consumption	Yes	195 (78.3)	7.16 ± 1.17	0.279
	No	55 (21.7)	6.64 ± 1.45	
Alcohol consumption	Yes	86 (34.5)	7.17 ± 1.03	0.301
	No	164 (65.5)	6.99 ± 1.36	
Self-assesment of health (global)	Very good	7 (2.8)	6.00 ± 1.63	0.755
	Good	1 (0.4)	6.00	
	Moderate	77 (30.8)	6.76 ± 1.41	
	Bad	161 (64.4)	7.25 ± 1.13	
	Very bad	4 (1.6)	7.25 ± 0.50	

**Table 2:** Health characteristics of cancer patients and social support score.

Variables		*N*° (%)	Social support score	*p*
The presence of a long-term illness	Yes	242 (96.8)	7.04 ± 1.25	0.315
	No	8 (3.2)	6.43 ± 1.39	
Activity restrictions	Serious restriction	98 (39.2)	7.37 ± 1.14	0.893
	Restriction	147 (58.8)	6.87 ± 1.28	
	Without restriction	5 (2.0)	6.60 ± 1.67	
Chronic disease/condition in addition to cancer in the previous 12 months	Without other diseases besides cancer	7 (2.8)	6.71 ± 1.79	0.751
	One	10 (4.0)	6.70 ± 1.57	
	Two	20 (8.0)	6.40 ± 1.39	
	Three	21 (8.4)	6.81 ± 1.40	
	Four	54 (21.6)	6.87 ± 1.36	
	Five	44 (17.6)	7.11 ± 1.31	
	More than five	94 (37.6)	7.40 ± 0.91	
Difficulties in performing daily activities	No difficulties	141 (56.4)	6.86 ± 1.36	0.652
	Less difficulties	85 (34.0)	7.28 ± 1.07	
	Great difficulties	22 (8.8)	7.32 ± 0.99	
	Unable to perform them	2 (0.8)	8.50 ± 0.71	
Help with activities	Yes	103 (41.2)	7.33 ± 0.95	0.904
	No	147 (58.8)	6.87 ± 1.40	
The need for more help	Yes	155 (62.0)	7.32 ± 1.12	
	No	95 (38.0)	6,62 ± 1,35	
Difficulties in doing housework	No difficulties	29 (11.6)	6.68 ± 1.28	0.789
	Less difficulties	146 (58.4)	6.93 ± 1.31	
	Great difficulties	68 (27.2)	7.44 ± 1.09	
	Unable to perform them	7 (2.8)	7.57 ± 0.53	
Help in the house	Yes	175 (70.0)	7.17 ± 1.13	0.312
	No	75 (30.0)	6.80 ± 1.48	
The need for additional help at home	Yes	203 (81.2)	7.15 ± 1.18	0.298
	No	47 (18.8)	6.65 ± 1.49	
The presence of body pain in the previous 4 weeks	Without pain	–	–	0.952
	Very weak	31 (12.4)	6.87 ± 1.61	
	Weak	33 (13.2)	6.60 ± 1.11	
	Moderate	124 (49.6)	7.05 ± 1.17	
	Strong	61 (24.4)	8.00	
	Very strong	1 (0.4)	7.41 ± 1.23	
Impact of pain on activities	Not at all	6 (2.4)	7.00 ± 1.54	0.897
	Low	48 (19.2)	6.75 ± 1.36	
	Moderate	118 (47.2)	6.91 ± 1.16	
	Strong	63 (25.2)	7.41 ± 1.25	
	Very strong	15 (6.0)	7.64 ± 1.01	
Hospital treatment in the last 12 months	Yes	184 (73.6)	7.09 ± 1.19	0.411
	No	66 (26.4)	6.95 ± 1.41	
Number of nights spent in hospital	Up to 5 days	56 (22.3)	6.75 ± 1.19	0.921
	Up to 10 days	40 (15.9)	6.85 ± 1.36	
	Up to 15 days	9 (3.6)	8.11 ± 0.78	
	Up to 20 days	7 (2.8)	8.00	
	>20 days	1 (0.4)	7.00	
	Do not know	138 (55.0)	7.25 ± 1.12	
Number of day hospital admissions	One	7 (2.8)	7.14 ± 1.21	0.903
	Two	11 (4.4)	7.09 ± 1.05	
	Three	19 (7.6)	7.32 ± 0.88	
	Four	3 (1.2)	7.00	
	Five	7 (2.8)	8.28 ± 1.38	
	Six	26 (10.4)	5.73 ± 1.25	
	Seven	2 (0.8)	7.50 ± 0.71	
	Ten	15 (6.0)	7.35 ± 0.92	
	Do not know	161 (64.1)	7.60 ± 1.05	
Stage of the disease	Patients in the first stage	165 (66.0)	7.03 ± 1.25	0.912
	Patients in the second stage	83 (33.2)	7.11 ± 1.29	
	Third stage of the disease	2 (0.8)	7.0 ± 0.0	
Operated and non-operated	Operated	138 (55.2)	6.99 ± 1.32	0.348
	Non-operated patients	112 (44.8)	7.14 ± 1.6	
Тype of therapy (chemo, radio, combined)	Chemotherapy	92 (36.8)	6.64 ± 1.21	0.963
	Radiotherapy	146 (58.4)	7.12 ± 1.1	
	Combined therapy	12 (4.8)	7.42 ± 1.25	

Breast cancer (28.3%), bronchial and lung cancer (7.2%) and prostate cancer (5.6%). were the most common cancers in the studied population. Statistical data processing revealed the existence of a significant difference in the social support score among patients suffering from different types of cancer (One-Way ANOVA, df (30) = 2.217, *p* = 0.001). The highest average values of the social support score were found in patients with pancreatic cancer (8.2) and bladder cancer (8.1) (Table 3).

**Table 3:** Social support score in patients with different cancers.

ICD mark	Localization	Percentage share in the study population	Social support score (*Ȳ*)	SD
C00	Lips	0.8	6.0	1.41
C10	Oropharynx	2.0	6.4	0.89
C17	Small intestine	1.6	8.0	1.41
C18	Colon	4.4	7.1	0.54
C19	Sigma and rectum	3.6	7.7	0.71
C20	Rectum	3.6	7.0	1.12
C21	Anus	5.2	7.6	1.12
C23	Gallbladder	1.2	8.0	1.0
C25.9	Pancreas	1.6	8.2	0.5
C30	Nasal cavity	0.4	8.0	–
C32	Larynx	2.4	6.5	0.55
C33	Trachea	0.8	7.0	0,0
C34	Bronchus and lungs	7.2	7.7	0.68
C43	Melanoma	3.2	6.0	1.85
C44	Skin	1.6	7.8	0.96
C45	Pleural mesothelioma	0.8	7.0	0.0
C50	Breast	28.3	6.6	1.37
C51	Vulva	0.8	7.0	1.41
C52	Vagina	1.2	6.0	1.73
C53.1	Exocervix	5.2	6.6	1.38
C54.3	Uterus	1.2	5.7	0,57
C56	Ovary	2.4	7.8	1.33
C61	Prostate	5.6	7.1	1.29
C62	Testicle	0.4	8.0	–
C64	Kidney	3.6	6.7	1.0
C67	Bladder	3.2	8.1	0.64
C70	Meninges	0.4	7.0	–
C73	Thyroid gland	2.0	7.4	1.34
C77	Lymph nodes	4.0	7.0	–
C81.9	Hodgkin	1.2	7.0	1.73
C90	Multiple myeloma	3.6	8.0	0.0

Data collected from the entire study population showed that bad social support was present in almost 90% of cases. Univariate and multivariate regression analysis showed a statistically significant influence of the following variables on the bad social support: education level, activity limitation, difficulties in performing daily activities, the impact of pain on the performance of activities, the need for additional help with activity, the need for help at home, unfulfilled needs for health care, means of information, anxiety score and depression score (Table 4).

**Table 4:** Odds ratios (ORs) with the corresponding 95% confidence intervals (CIs) for the association between of social support score and patient characteristics.

	Univariate model		Multivariate model	
	OR (95%CI)	*p*	OR (95%CI)	*p*
Age (years)	0.58 (0.06–1.02)	0.359	0.60 (0.31–1.22)	0.502
Gender				
Women	0.168 (0.782–1.128)	0.168	1.208 (0.413–3.533)	0.729
Men	1		1	
Marital status				
Unmarried	0.176 (0.028–1.128)	0.067	0.29 (0.002–0.575)	0.20
A widower/a widow	1.843 (0.520–6.532)	0.344	2.094 (0.522–8.404)	0.297
Divorced	0.824 (0.223–3.036)	0.771	0.552 (0.124–2.457)	0.436
Married	1		1	
Education level				
Elementary school or lower	0.083 (0.011–0.643)	0.017	0.018 (0.001–0.341)	0.007
High school	0.181 (0.022–1.487)	0.112	2.632 (0.005–1.286)	0.074
College	1		1	
Employment status				
Inactive	0.339 (0.042–2.730)	0.310	0.270 (0.20–3.671)	0.325
Unemployed	0.293 (0.034–2.546)	0.266	0.128 (0.009–1.877)	0.134
Pensioner	0.850 (0.090–8.043)	0.887	0.241(0.016–3.713)	0.308
Employed	1		1	
Self-assesment of health				
Bed	0.381 (0.048–3.017)	0.360	0.230 (0.024–2.171)	0.200
Average	0.343 (0.041–2.865)	0.323	0.211 (0.022–1.989)	0.174
Good	1		1	
Оperated				
Yes	1.10 (0.90–1.34)	0.347	0.24 (0.03–0.40)	0.868
No	1		1	
Тherapy				
Chemotherapy	0.838 (0.097–7.267)	0.873	1.089 (0.105–11.264)	0.943
Radiatherapy	0.739 (0.089–6.104)	0.779	0.849 (0.076–9.443)	0.894
Combinedtherapy	1		1	
Activity limitation				
Yes	0.47 (0.18–0.77)	0.002	0.54 (0.39–0.76)	0.071
No	1		1	
Difficulties in performing daily activities				
Yes	0.33 (0.11–0.55)	0.004	0.31 (0.03–0.60)	0.033
No				
The impact of pain on the performance of activities				
Yes	0.30 (0.14–0.47)	0.001	0.21 (0.09–0.33)	0.317
No	1		1	
The need for additional help with activity				
Yes	0.71 (0.39–1.02)	0.001	0.53 (0.46–0.87)	0.027
No	1		1	
The need for help at home				
Yes	0.37 (0.03–0.71)	0.032	0.30 (0.19–0.68)	0.044
No	1		1	
Unfulfilled needs for health care				
Yes	0.57 (0.31–0.84)	0.001	0.32 (0.03–0.62)	0.034
No	1		1	
Means of information				
Yes	0.51 (0.27–0.76)	0.001	0.44 (0.17–0.72)	0.001
No	1		1	
Anxiety score				
Yes	2.34 (1.44–3.24)	0.001	1.23 (0.98–1.37)	0.001
No	1		1	
Depression score				
Yes	2.05 (1.41–2.70)	0.001	1.8 (1.15–2.35)	0.001
No	1		1	

## Discussion

The concept of social support involves the willing or actual provision of relationships, information, advice, or assistance that enables a person to successfully cope with the day-to-day challenges of crisis in their personal life. Social support has a structural and a functional dimension. The structural dimension refers to the presence of social relationships, and the functional dimension refers to the various types of help provided by people within a person’s social network that one usually thinks of when one thinks of social support. In addition, there are other categories of functional measurements, such as emotional, instrumental, and counseling support (Ruiz-Rodríguez et al., 2022). Social support plays an important role in the daily life of cancer patients. Some studies have shown that social support contributes to coping, good mood, physical condition, emotional status, and overall quality of life while reducing disease-related stress (Usta, 2012).

Social support is very important in helping cancer patients alleviate the negative effects of cancer diagnosis and treatment and improve the consequences of cancer disease. An analysis of population diagnosed with cancer found that patients who experienced more social support had stronger mental health and quality of life (Taylor et al., 2007; Kadambi et al., 2020).

The results of our research indicate poor social support in patients with malignant diseases. Also, when viewed in relation to the sociodemographic and health characteristics of the subjects of our study, a low level of social support can be observed.

One study showed that married patients had a greater sense of hope and more social support than single patients (Ghazzawi et al., 2016). Another study that included cancer patients also found that patients who have a partner or are in a marriage have a better quality of life and more social support than single patients (Lavdaniti et al., 2017). This is supported by cancer patients’ statements that their spouse is the most important source of social support (Leung et al., 2014), which is contrary to the results of our study, where a bad score of social support was recorded for married respondents. Results obtained after analysing variables with social support dimensions corroborate findings of literature, in which men received more social support than women. The results showed that patients who had stronger social support had a greater sense of security and were less tired from chemotherapy (Karakoç and Yurtsever, 2010).

There is also a study that shows the connection between a lower level of social support and a significantly higher level of depression, suggesting that social support contributes to mental health and a higher quality of life (Eom et al., 2013). Studies have shown that patients with strong social support develop more optimistic feelings, which enable them to increase their confidence and hope that they can successfully fight and cure cancer (Kyriazidou et al., 2022). Studies examining the relationship between social support, depression, and quality of life in patients with various cancer diagnoses found that patients who did not have significant social support were more likely to suffer from depression, had lower functional abilities, and had a lower quality of life (Korotkin et al., 2019), which is in line with the results of our reaserch in a statistically significant influence of the anxiety and depression as a predictor the bad social support.

A study examining the types of social support patients considered important concluded that cancer patients most frequently expressed a desire for companionship, empathy, assistance with home care, information, equal treatment, and help to make appointments at healthcare facilities. Anxious patients were more likely to want companionship, and younger patients were more likely to want assistance with home care (Nausheen et al., 2009; Schroevers et al., 2010).

The results suggest that support from family and friends in the postdiagnosis period plays an important role in helping cancer patients develop a positive attitude toward cancer (Calderon et al., 2021).

A study examining was the associations between perceived social support and sociodemographic variables on coping showed that sociodemographic factors as age, education, and partnership status were associated with coping strategies. Hopelessness was more frequent in older people and lower educational level and single people. Support from family, friends, and partners was associated with a greater fighting spirit. In contrast, high psychological distress, anxiety and depression was associated with bad level social support (Faraci and Bottaro, 2021), which is in line with the results of our reaserch which also suggest the connection education, anxiety, depression and social support.

The value of social support is that it plays an important role in preventing and reducing anxiety and depression in cancer patients, contributes to mental health and a higher quality of life. Furthermore, social support has a direct impact on insecurity/uncertainty and the greater the support of family members and healthcare professionals, the lesser the uncertainty in relation to treatment and pathology. Twhen this support is received, patients develop a sense of importance in a social network, respond positively to challenges and adopt positive behavior, such as initiating or maintaining actions that promote wellbeing in their social circle and enhance the proposed treatment. Social support can come from bonds between people and groups, which include natural collaborators (family), informal groups (self-help) and formal and institutionalized groups, such as organizations for the sick, that can create the support networks of patients. This support refers to the help of others in case of need and also reflects access to healthcare services and actions from other people that help to solve practical activities. The acknowledgement that social support can help cancer patients to adapt and maintain a quality of life means accepting the need to identify the arrangement of a support network made available to users so that care can be planned and implemented with quality (Kolankiewicz et al., 2014).

Although several valuable findings were identified in the present study, we must acknowledge some limitations. First and foremost is that the nature of cross-sectional survey design limits the ability of establishing causality between the proposed variables. And thus, further study with longitudinal design will be necessary to prospectively clear the mechanism of social support. Second, the sample is not representative. Data were obtained from a specific population group, with its cultural, economic and social configurations which does not have the same characteristics and potentialities in all regions of Serbia. Third, we assessed social support, using self-reported questionnaires in the present study and the results may be inflated due to subjective bias from participants.

### Implications for practice

Contributions of this study that should be emphasized are that the dimensions that make up social support should be topics of concern for healthcare professionals. Developing countries, such as Serbia, do not possess sufficient resources due to which it would be possible to provide adequate chosocial support to cancer-affected patients along with their family members. Practitioners can enhance the benefits of social support programs through strengthening. This study enhanced our understanding on the association social support in cancer patients Another contribution of the study is the acknowledgement that some groups are more vulnerable than others, and that socioeconimc factors should be observed in order to identify them and help these groups overcome difficulties in the initial stages of cancer. This study can serve as a model for investigations on other groups patients and for the characterization of social support.

## Conclusion

Considering the significance and influence of social support on patients‘mental and physical health, it is necessary not only to conduct further research studies on the factors having influence on the level social support, but to implement various interventions for the the purpose of their promotion as well. In order to create adequate public health policies and strategies that are needed to improve social support it is essential to determine and expose different predictors of bad social support. Encouraging patients with malignant diseases to activate social and social engagement can help reduce anxiety and depressive symptoms, prevent suicidal ideation, improve cognitive and functional status and enable easier coping with the disease and psychosocial difficulties, which will result in a better quality of life for these patients.

## Data availability statement

The raw data supporting the conclusions of this article will be made available by the authors, without undue reservation.

## Ethics statement

The studies involving human participants were reviewed and approved by the Ethics Committee of the Health Center Trstenik, Central Serbia (No 759/1 of 26.06.2020). The patients/participants provided their written informed consent to participate in this study.

## Author contributions

SC, VlV, and SnR: design and writing manuscript. OM, JD, and SC conducted the statistical analyses, commented on the manuscript and contributed to the background, and discussion section. SnR, IV, KJ, MS, SD VlV, OD, GD, and OM contributed to investigation. SC, VeV, OM, JD, SC, SnR, SvR, IV, KJ, MS, SD, VlV, OD, GD, and OM: critical revision and approval of the final draft. All authors contributed to the article and approved the submitted version.

## Conflict of interest

The authors declare that the research was conducted in the absence of any commercial or financial relationships that could be construed as a potential conflict of interest.

## Publisher’s note

All claims expressed in this article are solely those of the authors and do not necessarily represent those of their affiliated organizations, or those of the publisher, the editors and the reviewers. Any product that may be evaluated in this article, or claim that may be made by its manufacturer, is not guaranteed or endorsed by the publisher.
